# Isolation, Purification and Quantification of Ginsenoside F_5_ and F_3_ Isomeric Compounds from Crude Extracts of Flower Buds of *Panax ginseng*

**DOI:** 10.3390/molecules21030315

**Published:** 2016-03-09

**Authors:** Ke-Ke Li, Fei Xu, Xiao-Jie Gong

**Affiliations:** College of Medical, Dalian University, Dalian 116622, China; xufei910324@163.com

**Keywords:** ginsenoside F_5_, ginsenoside F_3_, isomer, purification, quantification

## Abstract

In this paper, the isolation, purification and quantification of ginsenoside F_5_ and F_3_ isomeric compounds from crude extracts of flower buds of *Panax ginseng* (CEFBPG) was investigated by reversed-phase high-performance liquid chromatography (RP-HPLC) for the first time. The satisfied separation at analytical scale was achieved using a Zorbax Eclipse XDB C-18 column with a ternary mobile phase of acetonitrile–water–phosphoric acid (28:71:1) at a flow rate of 1.0 mL/min within 40 min. UV detection was set at 203 nm. Ginsenoside F_5_ and F_3_ was 4.21 mg and 5.13 mg in 1 g flower buds of *P. ginseng* (FBPG), respectively. The preparation of ginsenoside F_5_ and F_3_ at semi-preparative scale was performed by using a Daisogel C-18 column and gradient elution system of acetonitrile–water (32:68 → 28:72) at a flow rate of 10 mL/min with a sample load of 20–30 mg, and yielded ginsenosides in purity of more than 96%. Their structures were characterized by NMR and high resolution electrospray ionization mass spectrometry (HRESIMS). All the method validations showed acceptable limits. The results indicate a new source to obtain ginsenoside F_5_ and F_3_, and show that the method developed here appears to be reliable for simultaneously preparing them from CEFBPG.

## 1. Introduction

*Panax ginseng* (PG) C. A. Meyer, one of the ancient and famous herbal roots in China, has been used as a tonic and for the treatment of various diseases [1,2,3,4,5,6]. Biologically active constituents have been pursued extensively from the different parts of PG including roots, stems, leaves, berries and flowers, and many dammarane-type triterpene oligoglycosides have been identified as the principal ingredients [7,8,9]. Recently, the saponins in the flower buds of *P. ginseng* (FBPG) have been reported including various dammarane-type saponins, the same as in ginsenoside roots, even several new active compounds [10,11,12]. Ginseng flowers has been recorded to strengthen body, tonify Qi, and delay the process of aging in many Chinese medical literatures such as “Zhong Hua Ben Cao” and “Chinese Herbal Medicine”. Therefore, ginseng flowers could also function as a supplementary source of pharmacologically active ginsenosides [13]. However, PG often blossoms only a flower on a plant after three or four years of growing, and the flowers were much more precious than the leaves, even though both were renewable resources. Amazingly, quantitative analysis showed that the total saponins in flowers was about three times higher than roots, and some specific ginsenosides were even ten times higher, such as ginsenoside Re and Rd [14]. The amount of total saponins in FBPG was 5%–7% [15], much higher than the roots and leaves, so it was an ideal source for obtaining the specific and pure ginsenoside compounds.

**Figure 1:**
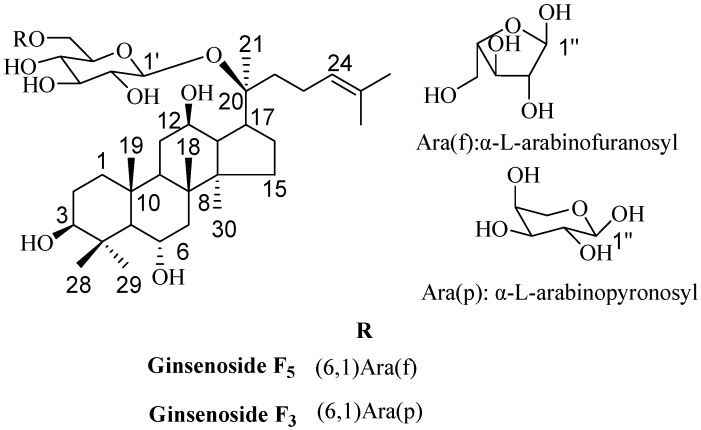
Chemical structures of ginsenoside F_5_ and F_3_.

Among the saponins of PG, the structure of ginsenoside F_5_ and F_3_ (Figure 1) was very similar, they were two isomeric compounds. The only difference was the configuration of the C-20 arabinosyl-(1→6)-β-d-glucopyranosyl; in ginsenoside F_5_ it was arabinofuranosyl, while in ginsenoside F_3_ it was arabinopyronosyl. According to the literature, ginsenoside F_5_ and F_3_ have been only found in the leaves of PG as minor ginsenoside [16], and this might partly affect the further scientific research. The reported activity studies showed that ginsenoside F_5_ remarkably inhibited the growth of HL-60 cells by the apoptosis pathway [12], and ginsenoside F_3_ had immunoenhancing activity by regulating productions and gene expressions of type 1 cytokines and type 2 cytokines in murine spleen cells [17]. However, if the two ginsenosides could be obtained easily and in high-quality, we might find new biological activities and their utility values would be increased.

The analytical approaches, including HPLC/UV [18], HPLC/evaporative light scattering detector (ELSD) [19] and LC-MS [20,21], have been developed to separate and identify ginsenosides of PG. Up to now, many efforts have been made to analyze the active saponins in PG, while studies on the preparation and quantification of the isomeric ginsenosides from crude extracts of flower buds of *P. ginseng* (CEFBPG) was rarely reported, and there were no publications on the isolation and preparation of ginsenoside F_5_ and F_3_ simultaneously. Moreover, producing highly pure saponins was beneficial to carrying out structure-activity relationship and other pharmaceutical studies.

Based on the above description, in this work, two isomeric compounds ginsenoside F_5_ and F_3_ were isolated from CEFBPG for the first time. We developed a specific RP-HPLC/UV method to prepare highly pure ginsenoside F_5_ and F_3_ from CEFBPG and determined their contents. The influences of several chromatographic conditions on the analysis and preparation were optimized. Chemical structure was characterized by NMR and electrospray ionization mass spectrometry (ESI-MS). The method validation was comprehensively performed including linearity, precision, recovery, limits of detection (LOD) and limits of quantification (LOQ).

## 2. Results and Discussion

### 2.1. Optimization of Analytical Method

#### 2.1.1. Influence of Mobile Phase Composition

**Figure 2:**
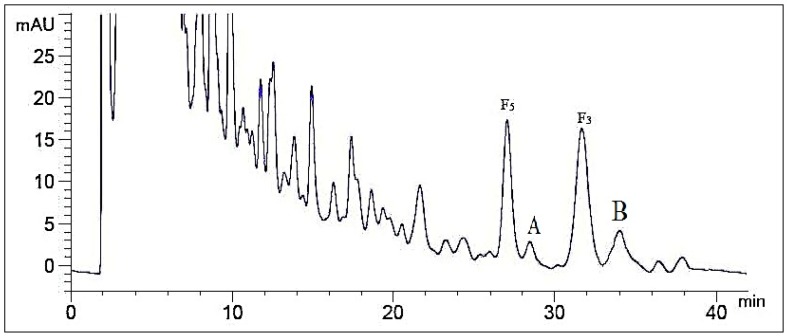
The chromatogram of CEFBPG (acetonitrile:water:phosphoric acid = 28:71:1).

Optimization of mobile phase composition in HPLC is investigated first, because this parameter plays a key role in resolution and sensitivity. In this section, the influence of mobile phase on separation effect was analysed in five different compositions: acetonitrile–water (30:70, *v*/*v*), acetonitrile–water–phosphoric acid (30:69:1), acetonitrile–water–phosphoric acid (28:71:1), methanol–water (60:40), methanol–water–phosphoric acid (60:39:1), as shown in Figure 2, Figures S1, S2, S7 and S8. Three determinations (*n* = 3) were carried out for each composition. The best separation was obtained with the ternary system of acetonitrile–water–phosphoric acid. The addition of phosphoric acid in mobile phase can help separation due to the presence of flavonoids in the CEFBPG, while a small amount of H_3_PO_4_ could reduce the ionization and lower the polarity of these compounds.

#### 2.1.2. Influence of Volume Ratios of Mobile Phase

Different volume ratios of acetonitrile–water–phosphoric acid (30:69:1, 30:69.5:0.5, 28:71:1, 28:71.5:0.5, 27:72:1) were assayed (*n* = 3) in order to achieve maximum sensitivity (Figure 2 and Figures S2–S6). A satisfactory separation was obtained with a mobile phase composed of acetonitrile–water–phosphoric acid (28:71:1). Increasing the increment of acetonitrile ratio to more than 28% led to overlappings of ginsenoside F_5_ and A, as well as ginsenoside F_3_ and B (compounds A and B were unknown). For lower acetonitrile ratio, the separation would take longer retention time. The typical chromatogram of the HPLC method was illustrated in Figure 2, which exhibited separation of ginsenoside F_5_, F_3_ and their adjacent compounds well.

### 2.2. Optimization of Semi-Preparative Method

From the results of analytical chromatography, the separation process was scaled up to preparative-scale chromatography. In this section, the preparative method was optimized for various parameters such as composition and volume ratios of mobile phase, flow rate, sample load. Three determinations (*n* = 3) were carried out for each solution. The eluent of ginsenoside F_5_ and F_3_ was collected over the period of each retention time for about 2 min.

#### 2.2.1. Optimization of Composition of Mobile Phase

**Figure 3:**
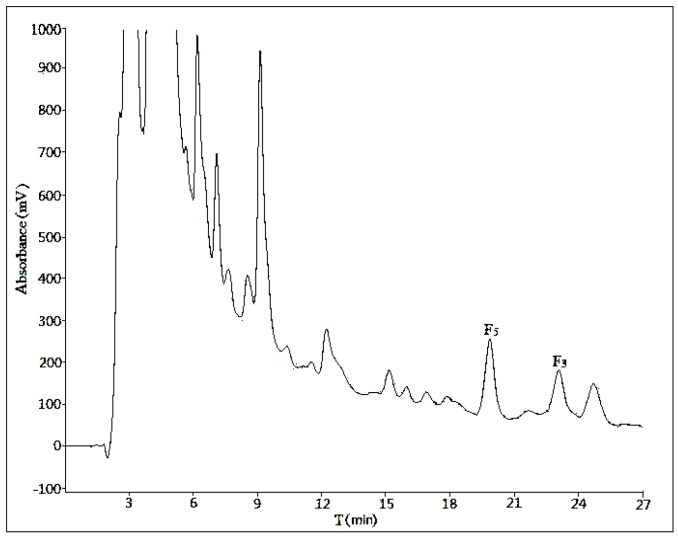
The semi-preparative chromatogram of CEFBPG with the mobile phase of acetonitrile:water (28:72) at room temperature and a flow rate of 10 mL/min.

**Figure 4:**
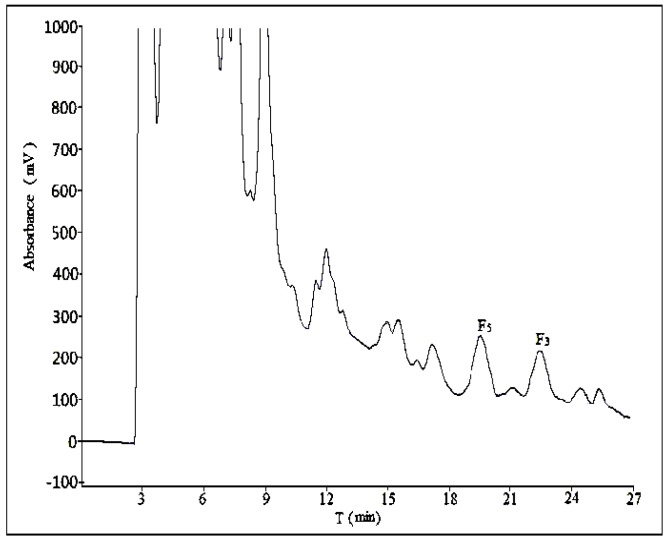
The semi-preparative chromatogram of CEFBPG with the mobile phase of acetonitrile:water:phosphoric acid (28:71:1) at room temperature and a flow rate of 10 mL/min.

**Figure 5:**
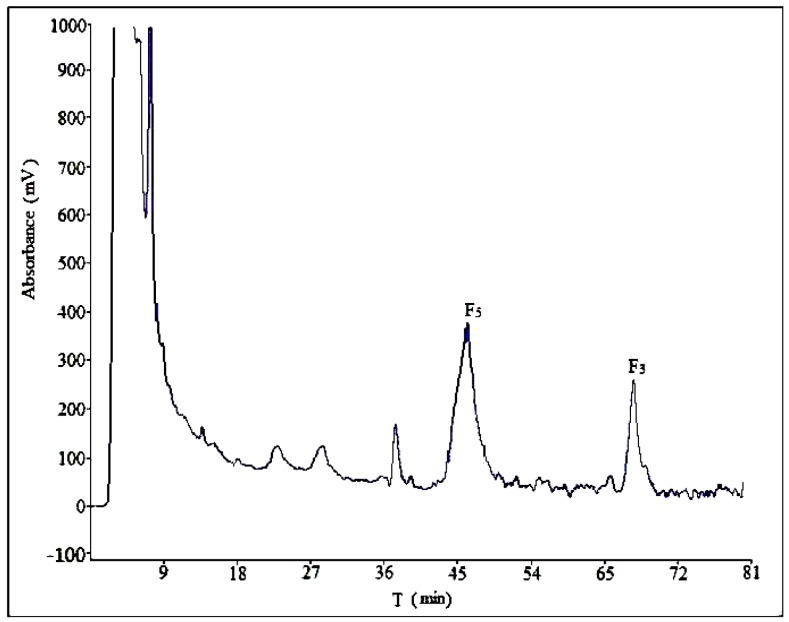
The semi-preparative chromatogram of CEFBPG with the mobile phase of methanol:water (58:42) at room temperature and a flow rate of 10 mL/min.

Three different compositions of mobile phase (acetonitrile:water = 28:72, acetonitrile:water:phosphoric acid = 28:71:1, methanol:water = 58:42) were tested at a flow rate of 10 mL/min with a sample load of 30 mg at room temperature. In Figure 3 and Figure 4, there was no obvious difference in separation effect whether the mobile phase was with or without phosphoric acid. In addition, in Figure 5, which used methanol–water as the mobile phase, the separation effect was very poor. Considering the shorter retention time and simplicity of the composition of mobile phase, we used a binary system of acetonitrile and water to achieve the best resolution.

#### 2.2.2. Optimization of Flow Rate

**Figure 6:**
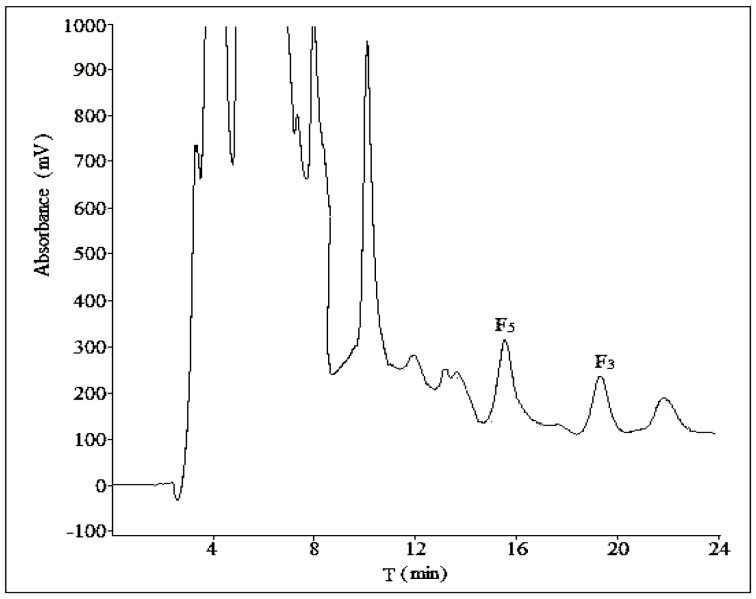
The semi-preparative chromatogram of CEFBPG with the mobile phase of acetonitrile:water (28:72) at room temperature and a flow rate of 15 mL/min.

An ideal flow rate of mobile phase can improve an effective separation and suitable retention time. As shown in Figure 3 and Figure 6, two different flow rates were tested (10 and 15 mL/min) with a mobile phase composed of acetonitrile and water. The higher flow rate generated a poorer separation effect of ginsenoside F_5_ and the unknown compound A, so the optimal flow rate was 10 mL/min.

#### 2.2.3. Optimization of Volume Ratios of Mobile Phase

**Figure 7:**
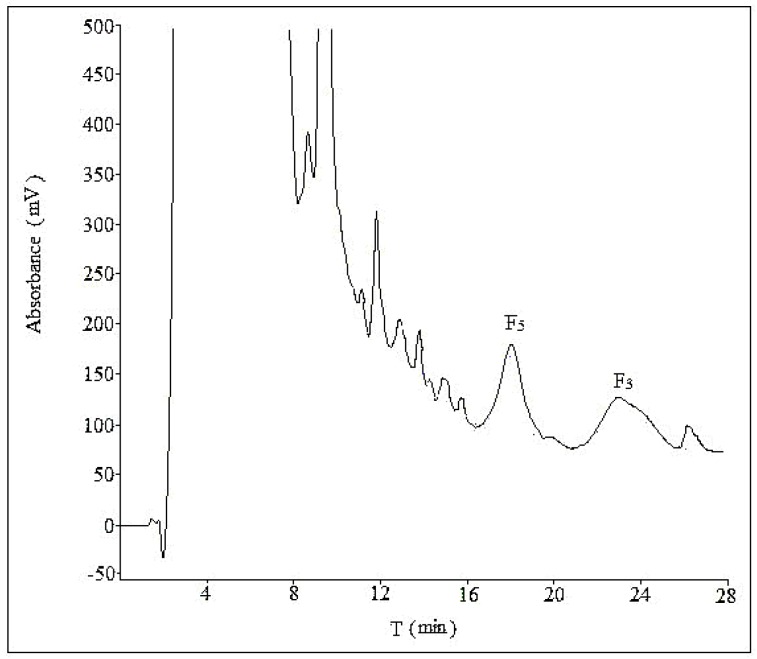
The semi-preparative chromatogram of CEFBPG with the mobile phase of acetonitrile:water (32:68) at room temperature and a flow rate of 10 mL/min.

**Figure 8:**
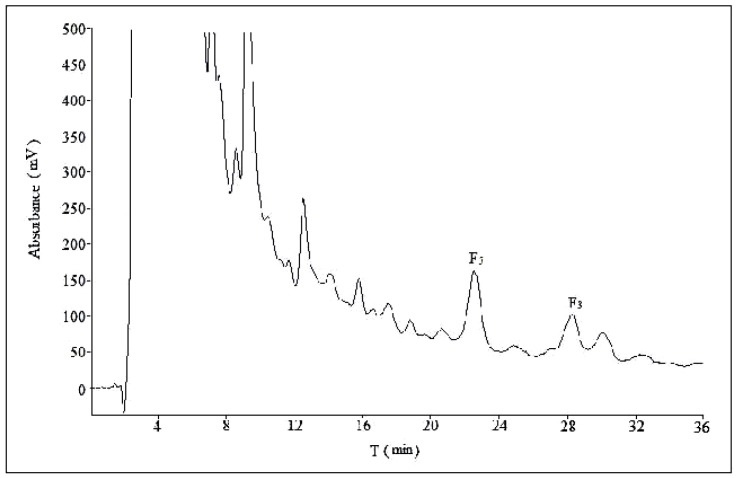
The semi-preparative chromatogram of CEFBPG with the mobile phase of acetonitrile:water 32:68 → 28:72 (15 min) at room temperature and a flow rate of 10 mL/min.

**Figure 9:**
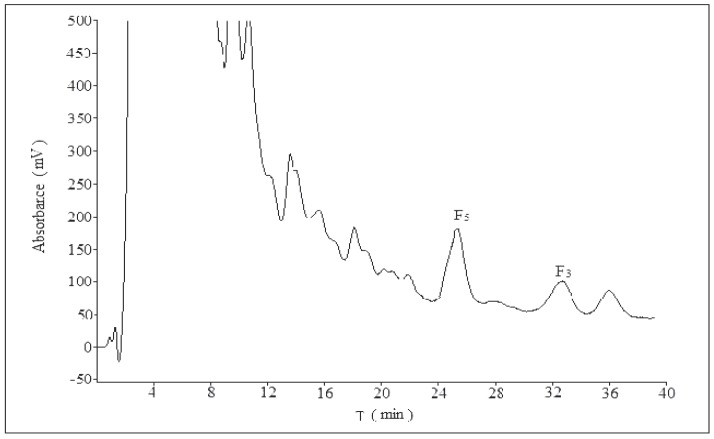
The semi-preparative chromatogram of CEFBPG with the mobile phase of acetonitrile:water 32:68 → 28:72 (10 min) at room temperature and a flow rate of 10 mL/min.

The volume ratios of the mobile phase play a key role in separation of ginsenosides in semi-preparative HPLC. Different volume ratios of acetonitrile:water 28:72; 32:68; 32:68 → 28:72 (0 min 28:72, 15 min 28:72); 32:68 → 28:72 (0 min 32:68, 10 min 28:72) were investigated at a flow rate of 10 mL/min (Figure 3, Figure 7, Figure 8 and Figure 9). The results shown in Table 1 indicated that the separation varied among four different volume ratios of mobile phase, especially for ginsenoside F_3_. A satisfactory separation was achieved with a gradient of acetonitrile–water (0 min 32:68, 10 min 28:72).

#### 2.2.4. Optimization of Sample Load

The effect of sample load was tested in the range from 10 to 50 mg at a flow rate of 10 mL/min using an acetonitrile–water system (32:68 → 28:72; 0 min 32:68, 10 min 28:72). The results shown in Table 2 indicated that the separation varied among different sample loads. Increasing the increment of the sample load from 10 to 30 mg resulted in little influence on the purity of ginsenoside F_5_ and F_3_. However, when the sample load was more than 30 mg, the purity of ginsenosides decreased markedly. The best separation was achieved at 20–30 mg, in which the semi-preparative chromatography gave a good purity and yielded relatively large amounts of ginsenosides.

### 2.3. Elucidation of Chemical Structures

Structures of ginsenoside F_5_ and F_3_ were confirmed by modern spectroscopic techniques, including NMR and MS. The NMR data were elucidated by comparing with those reported [22,23]. ^13^C-NMR data was shown in Table 3. The ^1^H-, ^13^C-NMR and MS spectra were shown in Supplementary Materials.

### 2.4. Method Validation

#### 2.4.1. Range and Linearity

Six concentrations of ginsenoside F_5_ and F_3_ methanol solution were injected in triplicate, and then the calibration curve was constructed by plotting the peak areas *versus* the concentration of each analyte. The calibration curves were *y* = 2650.5*x* + 29.279 for F_5_ and *y* = 1975.1*x* − 24.909 for F_3_, which showed good linear regression (*R*^2^ = 0.9994 for F_5_ and *R*^2^ = 0.9999 for F_3_) within 0.1–1.0 mg/mL.

#### 2.4.2. Accuracy and Precision

Accuracy and precision of the method were determined by analyzing two different concentrations (0.2, 0.4 mg/mL) within the calibration range and each concentration three times (*n* = 3). Accurate amounts of standard ginsenosides were added to an accurately weighed portion of FBPG and then prepared and analyzed as described above. The average recoveries were shown in Table 4, and both had an excellent accuracy recovery from 96.58% to 101.32% (*n* = 3) and an acceptable relative standard deviation (RSD) limits (<3%) for the analytes. The intra- and inter-day variability were evaluated for the precision of the method, the overall intra- and inter-day variations were less than 5%. These data demonstrated the acceptable precision of the method.

#### 2.4.3. Sensitivity

LOD and LOQ were used to represent the sensitivity of the method. The diluted standard solutions of ginsenoside F_5_ and F_3_ were further diluted with methanol to give a series of concentrations until signal-to-noise (S/N) ratio reached about three for determining the LOD, and S/N ratio was about 10 for determining the LOQ. In the method, the LOD and LOQ were determined to be 0.021 mg/mL and 0.052 mg/mL for ginsenoside F_5_, 0.011 mg/mL and 0.042 mg/mL for ginsenoside F_3_, respectively, indicating the method was extremely sensitive.

### 2.5. Quantitative Analysis of Ginsnoside F_5_ and F_3_ in FBPG

Two grams of FBPG sample was extracted in triplicate by reflux treatment with methanol (each 20 mL, *w*/*v* = 1:10). After the extracts was partitioned with dichloromethane and ethyl acetate, the unextracted residue was concentrated and dissolved in methanol with a 10 mL volumetric flask, then filtered through a 0.45 μm syringe filter, and it was ready for HPLC analysis. Each solution was analyzed three times, the content of ginsenoside F_5_ and F_3_ from Tonghua County was (4.21 ± 0.04) mg and (5.13 ± 0.03) mg in 1 g FBPG (*n* = 9), respectively.

### 2.6. Discussion

The reported methods of extraction and purification of ginsenosides F_5_ and F_3_ usually use aqueous ethanol or aqueous methanol as the extraction solvent, then followed by reverse-phase polystyrene gel or ordinary-phase silica gel column chromatography [22,23,24]. However, the aqueous solvent may lead to the presences of more large polar substances, such as oligoglycosides, polysaccharides and inorganic salt. Ginsenosides F_5_ and F_3_ are compounds with medium polarity and good solubility in methanol, so they can be extracted relatively completely in methanol with less needless components under this work. Meanwhile, the pre-purification with dichloromethane and ethyl acetate can remove the chemical constituents of low polarity, leading to less disturbances to the separation, thus this procedure can also provide simplified ways of purification. The similar method of pre-purification for ginsnosides can be seen in the studies of Kwon *et al.* [25] and Gu *et al.* [26]. In another study carried out by Magiera *et al.* [27], ginsenoside Re was separated and purified from ginseng bud by selective adsorption of active carbon before preparative HPLC. Although the adsorption ability of ginsenoside Re by active carbon is much weaker than any other kind of ginsenosides, there is more or less adsorbance for ginsenoside Re. In contrast, in our work made by the extraction with dichloromethane and ethyl acetate, there was no loss since ginsenosides F_5_ and F_3_ did not dissolve in the two solvents. The other applied pretreatment methods such as solid phase extraction [28,29] and centrifugation [30,31] were also adaptable to ginsenosides, but they were not very suitable for the large scale of ginsenoside preparation.

We used two different methods for the separation of ginsenosides F_5_ and F_3_ in analytical and semi-preparative experiments. The method of semi-preparation was derived from the improvement of analytical methods. Due to the different chromatographic column used and the large sample loads in semi-preparative experiments, a gradient elution of acetonitrile-water could obtain a satisfactory separation with shorter retention time. As to the addition of phosphoric acid to the mobile phase, it showed no obvious difference in the separation effect of semi-preparative experiments (Figure 3 and Figure 4), so the optimization result of composition of mobile phase was performed without phosphoric acid. The volume ratios of acetonitrile in mobile phase played an important role in the separation effect of the two ginsenosides, and it should be no more than 28% (Figure 7 and Figures S1–S3). In the reported study on the purification of ginsenoside Re from ginseng bud [27], the volume ratios of acetonitrile in mobile phase was also one of the important influence factors for the separation conditions.

The time-saving and decrease in the total consumption of mobile phase implies a reduction in analysis cost. Compared with the preparative HPLC/UV for ginsenoside Re [27] and the semi-preparative HPLC/ELSD for ginsenosides Rb_3_ and Rc [32], our semi-preparative HPLC/UV method offered a threefold decrease in retention time.

Based on the extraction of FBPG with methanol and followed by the solvent extraction with dichloromethane and ethyl acetate, we obtained an efficient performance by the validated semi-preparative HPLC method, as it achieved the separation and purification of two isomeric compounds ginsenoside F_5_ and F_3_ and got high-quality monomers.

## 3. Materials and Methods 

### 3.1. Reagents, Materials and Preparation of CEFBPG

The FBPG were collected at Tonghua County (Jilin, China) in 2014 and were taxonomically identified by one of us (Xiao-Jie Gong). The air-dried material (2.5 kg) was powdered in a pulverizer (XS-10B, Longxin, China) and passed through an 80 mesh sieve. The extraction of saponins from the flower buds of PG was performed three times by reflux treatment with methanol (each 25 L, *w*/*v* = 1:10). The extracts were combined and concentrated to small volume (3 L), which was partitioned with dichloromethane and ethyl acetate in sequence. Then, the unextracted residue residue was concentrated and the CEFBPG was obtained. The reference standards ginsenoside F_3_ and F_5_ were purchased from National Institutes for Food and Drug Control. All reagents were of chromatographic grade, except the phosphoric acid, which was of analytical grade.

### 3.2. Chromatographic Analysis

The analytical HPLC system was an Agilent Series 1260 (Agilent Technologies, Santa Clara, CA, USA) liquid chromatography system, equipped with a vacuum degasser, a quaternary pump, a column oven, a 7125i Reodyne Model manual injector with a 20 μL loop and a diode array detector (DAD). The chromatographic separation of the compounds was achieved with a Agilent Zorbax Eclipse XDB C-18 (5 μm; 4.6 mm i.d. × 250 mm, St. Louis, MO, USA) column. UV detection of samples was performed at a wavelength of 203 nm with a flow rate of 1.0 mL/min.

The semi-preparative HPLC was performed on a CXTH HPLC system (Chuang Xin Tong Heng Sci. Technol. Co. Ltd., Beijing, China) equipped with two P3050 pumps, a UV-3000 ultraviolet-visible detector (Chuang Xin Tong Heng Sci. Technol. Co. Ltd., Beijing, China), and a 7125i Reodyne Model manual injector with a 5 mL loop, using a Daisogel C-18 column (5 μm, 20 mm i.d. × 250 mm, Mcirowants, Suzhou, China). The data acquisition system was CXTH-3000 chromatography workstation (Chuang Xin Tong Heng Sci. Technol. Co. Ltd., Beijing, China).

### 3.3. Semi-Preparative Liquid Chromatography Separation

The CEFBPG was dissolved in the mobile phase of semi-preparative liquid chromatography, then filtered through a 0.45 μm syringe filter (Whatman, Brentford, Middlesex, UK). The resulted clear yellow solution is directly injected into the column by a 7125i Rheodyne injector. The fractions containing individual ginsenoside were collected respectively, and then combined and concentrated under vacuum. Ginsenoside F_5_ and F_3_ were finally obtained as white powder.

### 3.4. Structure Characterization of Ginsenosides

The isolated ginsenosides were characterized by NMR and electrospray ionization mass spectrometry (ESI-MS). NMR spectra were measured on a Bruker DRX500 spectrometer (Bruker S.A., Wissembourg, France) with tetramethyl silicane (TMS) as internal standard and pyridine-*d*_5_ as the solvent. Chemical shifts are reported in δ (ppm). The mass detection was performed on an Orbitrap Elite mass spectrometer (Thermo Scientific, Bremen, Germany) equipped with an ESI source. Melting point was recorded on a X-4 microscopic melting point meter (uncorrected) (Maisiqi High-tech Co. Ltd., Beijing, China).

Ginsenoside F_5_: white powder. m.p.: 186–187 °C. ESI-MS (*m*/*z*): 793.4648 [M + Na]^+^ (calcd C_41_H_70_O_13_Na 793.4714), 459.3794 [M − GlcAra(f) + H]^+^, 441.3690 [M − GlcAra(f) − H_2_O + H]^+^, 423.3588 [M − GlcAra(f) − 2H_2_O + H]^+^, 405.3487 [M − GlcAra(f) − 3H_2_O + H]^+^, 335.0914 [GlcAra(f) + Na]^+^. 

Ginsenoside F_3_: white powder. m.p.: 192–194 °C. ESI-MS (*m*/*z*): 793.4674 [M + Na]^+^ (calcd C_41_H_70_O_13_Na 793.4714), 459.3810 [M − GlcAra(p) + H]^+^, 441.3704 [M − GlcAra(p) − H_2_O + H]^+^, 423.3600 [M − GlcAra(p) − 2H_2_O + H]^+^, 405.3499 [M − GlcAra(p) − 3H_2_O + H]^+^, 335.0918 [GlcAra(p) + Na]^+^.

### 3.5. Validation

The proposed HPLC method was validated for various parameters such as linearity, range, precision, recovery, LOD and LOQ. Calibration curve with six concentration levels of standard solutions of each compound was constructed in the range of 0.1–1.0 mg/mL (*n* = 3). Precision was evaluated on both intra- and inter-day by analyzing two standard solutions (0.2, 0.4 mg/mL) within one day and on six different days (*n* = 6), respectively. Recovery of the method was performed at two different concentrations of 0.2, 0.4 mg/mL. LOD and LOQ were estimated from the plots as 3 and 10 times of the noise level, respectively.

## 4. Conclusions

In this study, a simple and reliable RP-HPLC method has been developed for the separation and quantification of two isomeric compounds ginsenoside F_5_ and F_3_ in FBPG for the first time. We comprehensively optimized parameters to be in the acceptable limits for the analytical method. Then, the semi-preparative RP-HPLC/UV method was developed on the basis of analytical method and yielded highly pure ginsenoside F_5_ and F_3_ from CEFBPG for the first time. This suggests that the active ginsenoside F_5_ and F_3_ could be obtained from FBPG by our extraction and purification method. These results are definitely helpful to expand the source of the two ginsenosides and provide a scientific basis for finding out the components that are responsible for total saponins of FBPG’s pharmacological effects.

## Figures and Tables

**Table 1:** The influence of the volume ratios of mobile phase on the separation effect of crude extracts of flower buds of *P. ginseng* (CEFBPG).

Test No.	Volume Ratios of Moble Phase (Acetonitrile–Water) (*n* = 3)	Peak Purity of Ginsenoside F_5_ (%) ^a,b^	RSD (%) (Ginsenoside F_5_)	Peak Purity of Ginsenoside F_3_ (%) ^a,b^	RSD (%) (Ginsenoside F_3_)
1	28:72	96.55 ± 1.18	1.22	94.69 ± 1.25	1.32
2	32:68	96.19 ± 1.35	1.41	90.47 ± 2.45	2.71
3	32:68 → 28:72 (15 min)	95.87 ± 0.87	0.91	94.09 ± 1.02	1.08
4	32:68 → 28:72 (10 min)	96.96 ± 1.04	1.08	97.18 ± 1.27	1.30

^a^ Peak purity: calculated by the peak area normalization method; ^b^ Results of peak purity are the means ± SD of four independent experiments performed in triplicate.

**Table 2:** The influence of the sample load on the separation effect of CEFBPG.

Sample Load (mg)	Peak Purity of Ginsenoside F_5_ (%) ^a,b^	RSD (%) (Ginsenoside F_5_)	Peak Purity of Ginsenoside F_3_ (%) ^a,b^	RSD (%) (Ginsenoside F_3_)
10.2	97.18 ± 1.64	1.68	97.55 ± 2.11	2.16
15.6	96.50 ± 1.22	1.27	98.20 ± 1.38	1.40
18.4	97.21 ± 0.85	0.87	97.42 ± 1.94	1.99
21.1	96.88 ± 1.79	1.85	97.29 ± 0.92	0.95
28.8	97.43 ± 2.15	2.21	97.12 ± 0.89	0.92
30.5	96.14 ± 1.81	1.89	96.88 ± 1.55	1.59
32.8	95.26 ± 2.84	2.98	94.84 ± 1.05	1.11
43.5	93.17 ± 1.36	1.46	92.90 ± 1.56	1.68

^a^ Peak purity: calculated by the peak area normalization method; ^b^ Results of peak purity are the means ± SD of eight independent experiments performed in triplicate.

**Table 3:** ^13^C-NMR data of ginsenoside F_5_ and F_3_ in pyridine-*d*_5_.

No.	Ginsenoside F_5_	Ginsenoside F_3_	No.	Ginsenoside F_5_	Ginsenoside F_3_
1	39.4	39.5	22	36.2	36.3
2	28.2	28.2	23	23.2	23.3
3	78.6	78.6	24	126.1	126.0
4	40.4	40.4	25	131.1	131.3
5	61.8	61.8	26	25.8	25.9
6	67.8	67.9	27	17.9	18.0
7	47.5	47.5	28	32.0	32.1
8	41.3	41.3	29	16.5	16.6
9	50.0	50.0	30	17.5	17.7
10	39.4	39.5	1′	98.1	98.2
11	30.8	30.9	2′	75.1	75.0
12	70.3	70.4	3′	79.3	79.2
13	49.1	49.2	4′	72.2	72.2
14	51.4	51.5	5′	76.6	76.8
15	30.9	30.9	6′	68.6	69.3
16	26.7	26.8	1′′	110.2	104.7
17	51.7	51.8	2′′	83.4	71.9
18	17.7	18.0	3′′	78.9	74.2
19	17.5	17.5	4′′	86.1	68.6
20	83.3	83.6	5′′	62.7	65.7
21	22.4	22.4			

**Table 4:** Recovery of ginsenoside F_5_ and F_3_ (*n* = 3).

Ginsenosides	Original (μg)	Spiked (μg)	Found (μg) (Mean ± SD)	Recovery (%) ^a^	RSD (%)
F_5_	210.6	200	405.9 ± 3.70	97.10	1.04
		400	596.6 ± 3.36	96.58	0.84
F_3_	252.4	200	452.2 ± 4.46	99.91	2.23
		400	657.6 ± 4.38	101.32	1.09

^a^ Recovery (%) = 100 × (amount found − original amount)/amount spiked.

## Supplementary Materials

Supplementary materials can be accessed at: http://www.mdpi.com/1420-3049/21/3/315/s1.

## Abbreviations

Crude extracts of flower buds of *Panax ginseng* (CEFBPG); flower buds of *Panax ginseng* (FBPG); high-performance liquid chromatography coupled with ultra violet detector (HPLC/UV); high-performance liquid chromatography coupled with evaporative light scattering detector (HPLC/ELSD); high resolution electrospray ionization mass spectrometry (HR-ESIMS); liquid chromatography coupled to mass spectrometry (LC-MS); limits of detection (LOD); limits of quantification (LOQ); nuclear magnetic resonance (NMR); relative standard deviation (RSD); reversed-phase high-performance liquid chromatography (RP-HPLC); signal-to-noise (S/N).

## References

[1] Lee C.H., Kim J.H. (2014). A review on the medicinal potentials of ginseng and ginsenosides on cardiovascular diseases. J. Ginseng Res..

[2] Song J.H., Choi H.J., Song H.H., Hong E.H., Lee B.R., Oh S.R., Choi K., Yeo S.G., Lee Y.P., Cho S. (2014). Antiviral activity of ginsenosides against coxsackievirus B3, enterovirus 71, and human rhinovirus 3. J. Ginseng Res..

[3] Li K.K., Gong X.J. (2015). A review on the medicinal potentials of Panax ginseng saponins in diabetes mellitus. RSC Adv..

[4] Wong A.S.T., Che C.M., Leung K.W. (2015). Recent advances in ginseng as cancer therapeutics: A functional and mechanistic. Nat. Prod. Rep..

[5] Park J.D., Rhee D.K., Lee Y.H. (2005). Biological activities and chemistry of saponins from *Panax ginseng* C.A. Meyer. Phytochem. Rev..

[6] Lee S.H., Jung B.H., Kim S.Y., Lee E.H., Chung B.C. (2006). The antistress effect of ginseng total saponin and ginsenoside Rg3 and Rb1 evaluated by brain polyamine level under immobilization stress. Pharmacol. Res..

[7] Li K.K., Yao C.M., Yang X.W. (2012). New dammarane-type triterpene saponins from the stems and leaves of *Panax ginseng* and their cytotoxicity on HL-60 cells. Planta Med..

[8] Li K.K., Yang X.B., Yang X.W., Liu J.X., Gong X.J. (2012). New triterpenoids from the stems and leaves of *Panax ginseng*. Fitoterapia.

[9] Yang W.Z., Hu Y., Wu W.Y., Ye M., Guo D.A. (2014). Saponins in the genus *Panax* L. (Araliaceae): A systematic review of their chemical diversity. Phytochemistry.

[10] Yoshikawa M., Sugimoto S., Nakamura S., Sakumae H., Matsuda H. (2007). Medicinal flowers. XVI. New dammarane-type triterpene tetraglycosides and gastroprotective principles from flower buds of *Panax ginseng*. Chem. Pharm. Bull..

[11] Tung N.H., Song G.Y., Nhiem N.X., Ding Y., Tai B.H., Jin L.G., Lim C.M., Hyun J.W., Park C.J., Kang H.K. (2010). Dammarane-type saponins from the flower buds of *Panax ginseng* and their intracellular radical scavenging capacity. J. Agric. Food Chem..

[12] Nguyen H.T., Song G.Y., Kim J.A., Hyun J.H., Kang H.K., Kim Y.H. (2010). Dammarane-type saponins from the flower buds of *Panax ginseng* and their effects on human leukemia cells. Bioorg. Med. Chem. Lett..

[13] Cho K., Song S.B., Tung N.H., Kim K.E., Kim Y.H. (2014). Inhibition of TNF-a-mediated NF-kB transcriptional activity by dammarane-type ginsenosides from steamed flower buds of *Panax ginseng* in HepG2 and SK-Hep1 cells. Biomol. Ther..

[14] Sugimoto S., Nakamura S., Matsuda H., Kitagawa N., Yoshikawa M. (2009). Chemical constituents from seeds of *Panax ginseng*: Structure of new dammarane-type triterpene ketone, panaxadione, and HPLC comparisons of seeds and flesh. Chem. Pharm. Bull..

[15] Ko S.K., Cho O.S., Bae H.M., Im B.O., Lee O.H., Lee B.Y. (2011). Quantitative analysis of ginsenosides composition in flower buds of various ginseng plants. J. Korean Soc. Appl. Biol. Chem..

[16] Tran T.L., Kim Y.R., Yang J.L., Oh D.R., Dao T.T., Oh W.K. (2014). Dammarane triterpenes from the leaves of *Panax ginseng* enhance cellular immunity. Bioorg. Med. Chem..

[17] Yu J.L., Dou D.Q., Chen X.H., Yang H.Z., Guo N., Cheng G.F. (2004). Immunoenhancing activity of protopanaxatriol-type ginsenoside-F3 in murine spleen cells. Acta Pharmacol. Sin..

[18] Liu G.Y., Zhou H.Y., Lu J., Zhu N., Gui M.Y., Jin Y.R., Zhang Y.H., Wang X., Li X.W. (2009). Determination of saponins in leaf of *Panax ginseng* C.A. Mey. by high performance liquid chromatography. Chem. Res. Chin. Univ..

[19] Kim S.N., Ha Y.W., Shin H., Son S.H., Wu S.J., Kim Y.S. (2007). Simultaneous quantification of 14 ginsenosides in *Panax ginseng* C. A. Meyer (Korean red ginseng) by HPLC-ELSD and its application to quality control. J. Pharm. Biomed. Anal..

[20] Fuzzati N., Gabetta B., Jayakar K., Pace R., Peterlongo F. (1999). Liquid chromatography-electrospray mass spectrometric identification of ginsenosides in *Panax giseng* roots. J. Chromatogr. A.

[21] Miao X.S., Metcalfe C.D., Hao C., March R.E. (2002). Electrospray ionization mass spectrometry of ginsenosides. J. Mass Spectrom..

[22] Dou D.Q., Chen Y.J., Ma Z.Z., Wen Y., Weng M.H., Pei Y.P., Wang Z.X., Kawai H., Fukushima H., Murakami Y. (1996). A novel minor saponin from the leaves of *Panax ginseng* C.A. Meyer. J. Chin. Pharm. Sci..

[23] Dou D.Q., Wen Y., Pei Y.P., Chen Y.J., Ma Z.Z. (1997). Studies on the minor saponins from leaves of *Panax ginseng* C.A. Meyer. Zhongguo Zhong Yao Za Zhi.

[24] Yoshizaki K., Devkota H.P., Yahara S. (2013). Four new triterpenoid saponins from the leaves of *Panax japonicus* grown in southern Miyazaki Prefecture (4). Chem. Pharm. Bull..

[25] Kwon S.W., Han S.B., Park I.H., Kim J.M., Park M.K., Park J.H. (2001). Liquid chromatographic determination of less polar ginsenosides in processed ginseng. J. Chromatogr. A.

[26] Gu Y., Wang G., Sun J., Xie H., Jia Y. (2006). Development of a sensitive LC-ESI-MS assay for 20(*R*)-ginsenoside Rh_2_ and its pharmacokinetic application in dogs: A case for the influence of micronization on traditional Chinese medicine. Intern. J. Mass Spectrom..

[27] Kuang P., Wang G., Yuan Q., Liang H. (2012). Separation and purification of ginsenoside Re from ginseng bud by selective adsorption of active carbon and preparative high-performance liquid chromatography. Nat. Prod. Res..

[28] Sun B.S., Gu L.J., Fang Z.M., Wang C.Y., Wang Z., Lee M.R., Li Z., Li J.J., Sung C.K. (2009). Simultaneous quantification of 19 ginsenosides in black ginseng developed from *Panax ginseng* by HPLC-ELSD. J. Pharm. Biomed. Anal..

[29] Wang C.Z., Wu J.A., McEntee E., Yuan C.S. (2006). Saponins composition in American ginseng leaf and berry assayed by high-performance liquid chromatography. J. Agric. Food Chem..

[30] Chen Y., Zhao Z., Chen H., Yi T., Qin M., Liang Z. (2015). Chemical differentiation and quality evaluation of commercial Asian and American ginsengs based on a UHPLC-QTOF/MS/MS metabolomics approach. Phytochem. Anal..

[31] Wang J., Liu C.M., Li L., Bai H.L. (2011). Isolation of four high-purity dammarane saponins from extract of *Panax notoginseng* by centrifugal partition chromatography coupled with evaporative light scattering detection in one operation. Phytochem. Anal..

[32] Liu C., Han J., Duan Y., Huang X., Wang H. (2007). Purification and quantification of ginsenoside Rb_3_ and Rc from crude extracts of caudexes and leaves of *Panax notoginseng*. Sep. Purif. Technol..

