# Supplementary material for: Isolation, Purification and Quantification of Ginsenoside F5 and F3 Isomeric Compounds from Crude Extracts of Flower Buds of Panax ginseng
Source: Molecules. 2016 Mar 9;21(3):315. doi: 10.3390/molecules21030315 (PMC6273083; doi:10.3390/molecules21030315)
Supplement: Supplementary file 1 [file molecules-21-00315-s001.pdf]

# Supplementary Materials: Isolation, Purification and Quantification of Ginsenoside $F_5$ and $F_3$ Isomeric Compounds from Crude Extracts of Flower Buds of *Panax ginseng*

**Ke-Ke Li, Fei Xu and Xiao-Jie Gong**

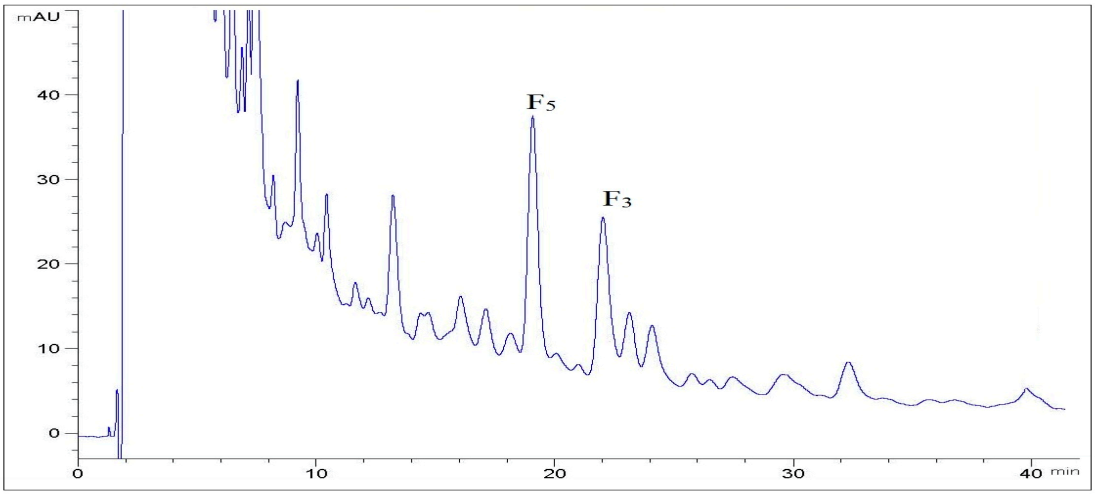


**Figure S1.** The chromatogram of CEFBPG (acetonitrile–water = 30:70).

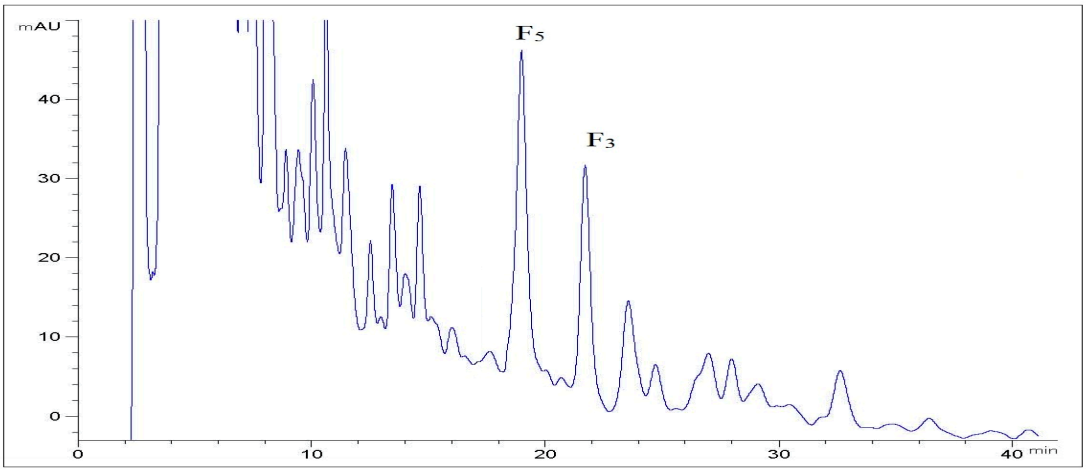


**Figure S2.** The chromatogram of CEFBPG (acetonitrile–water–phosphoric acid = 30:69:1).

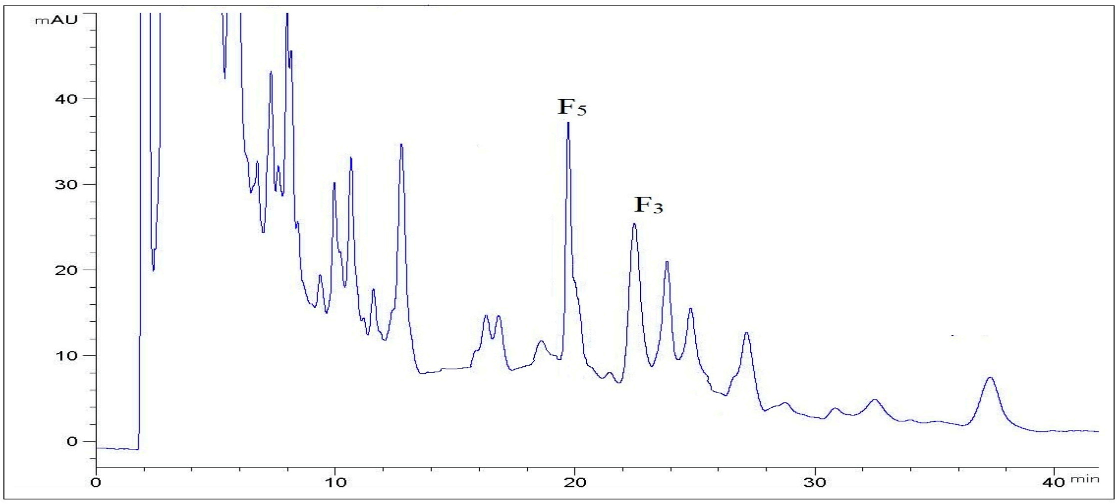


**Figure S3.** The chromatogram of CEFBPG (acetonitrile–water–phosphoric acid = 30:69.5:0.5).

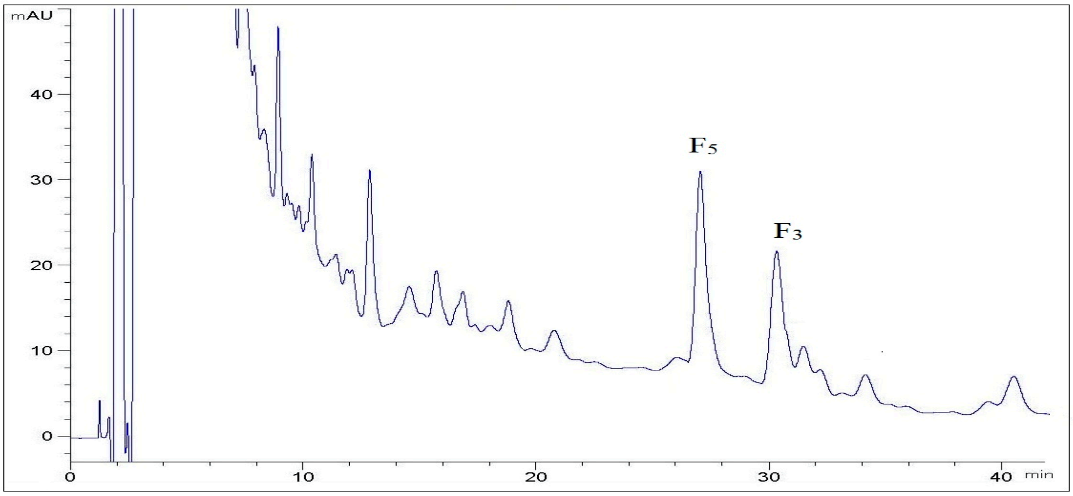


**Figure S4.** The chromatogram of CEFBPG (acetonitrile–water = 28:72).

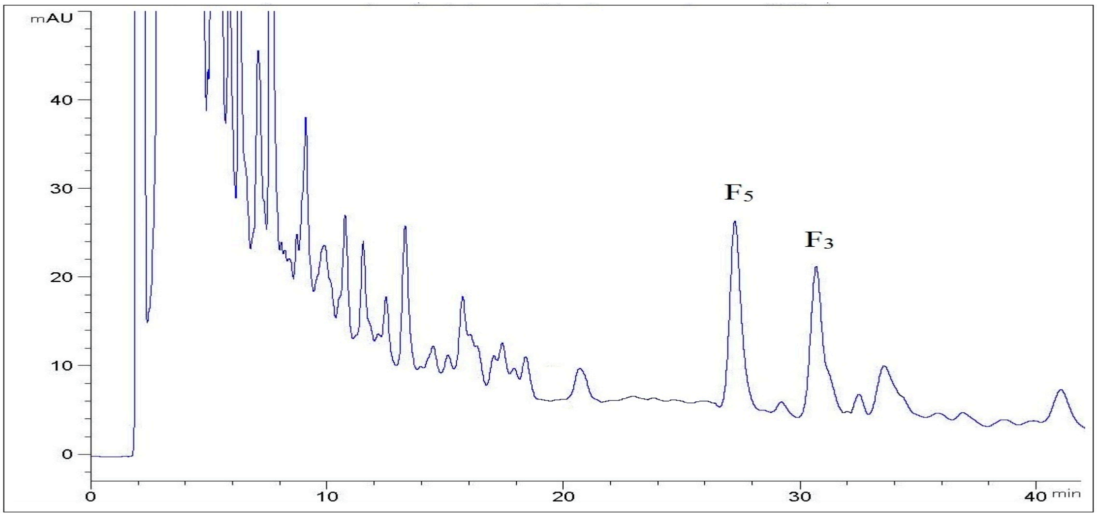


**Figure S5.** The chromatogram of CEFBPG (acetonitrile–water–phosphoric acid = 28:71.5:0.5).

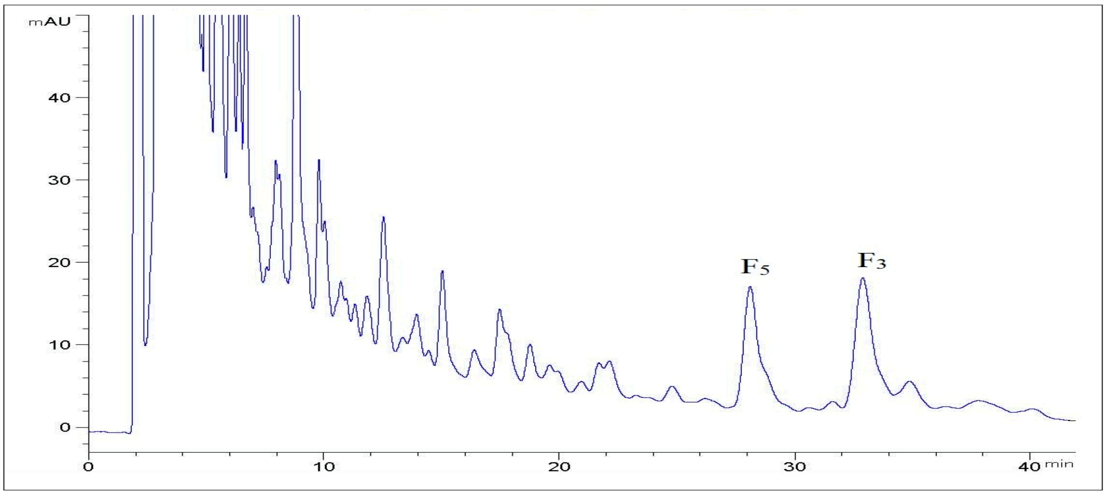


**Figure S6.** The chromatogram of CEFBPG (acetonitrile–water–phosphoric acid = 27:72:1).

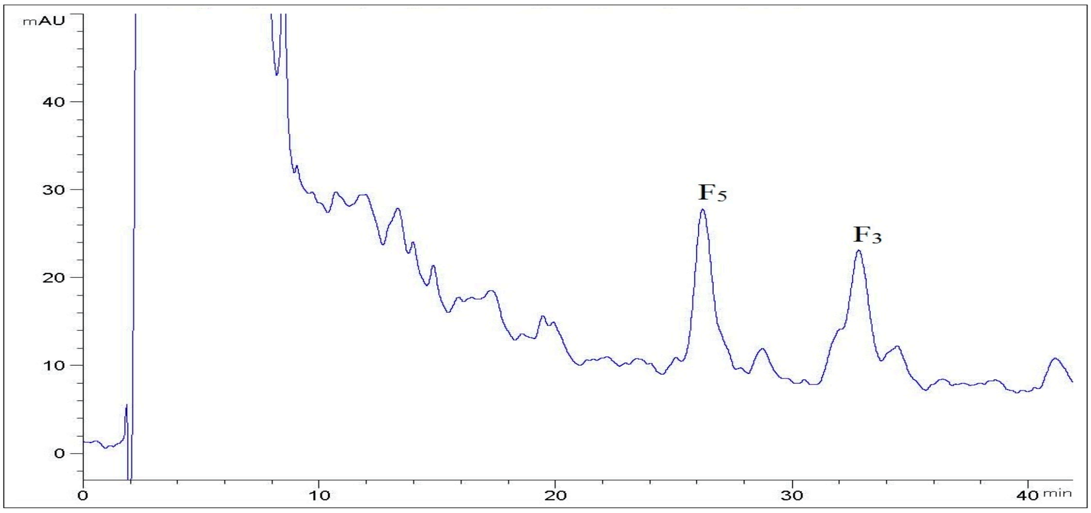


**Figure S7.** The chromatogram of CEFBPG (methanol–water = 60:40).

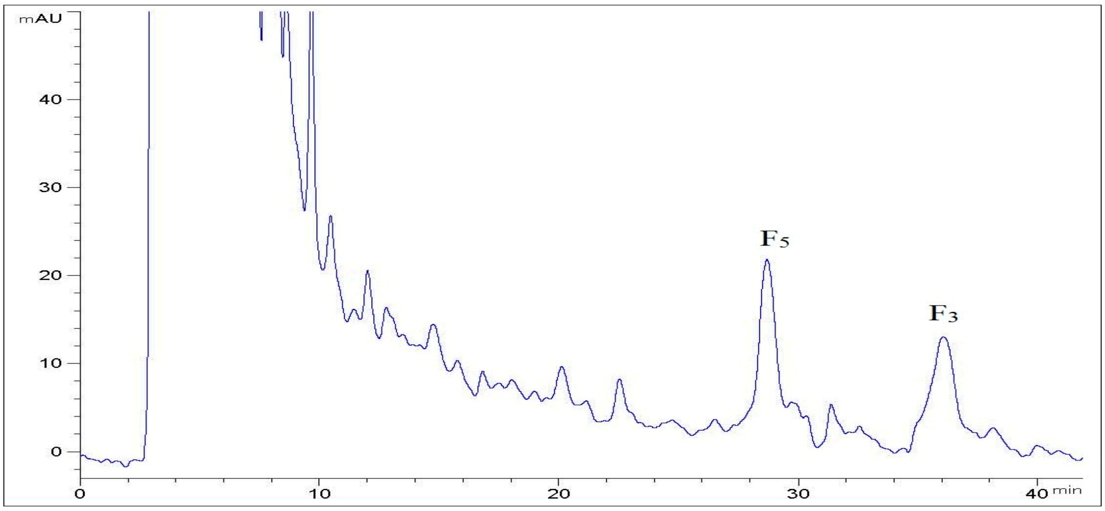


**Figure S8.** The chromatogram of CEFBPG (methanol–water–phosphoric acid = 60:39:1).

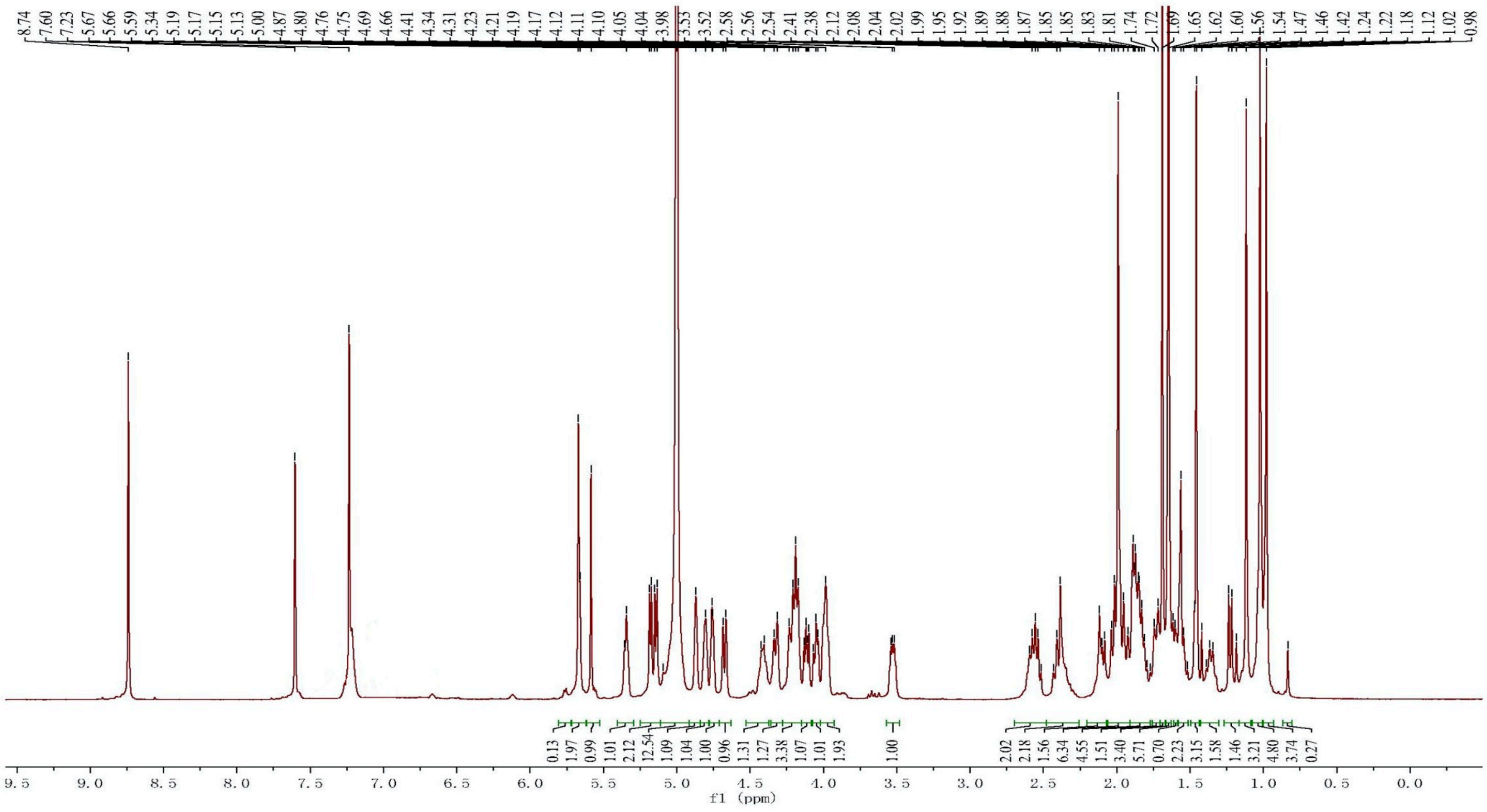


**Figure S9.** $^1$H-NMR spectrum of ginsenoside $F_5$ in pyridine-$d_5$.

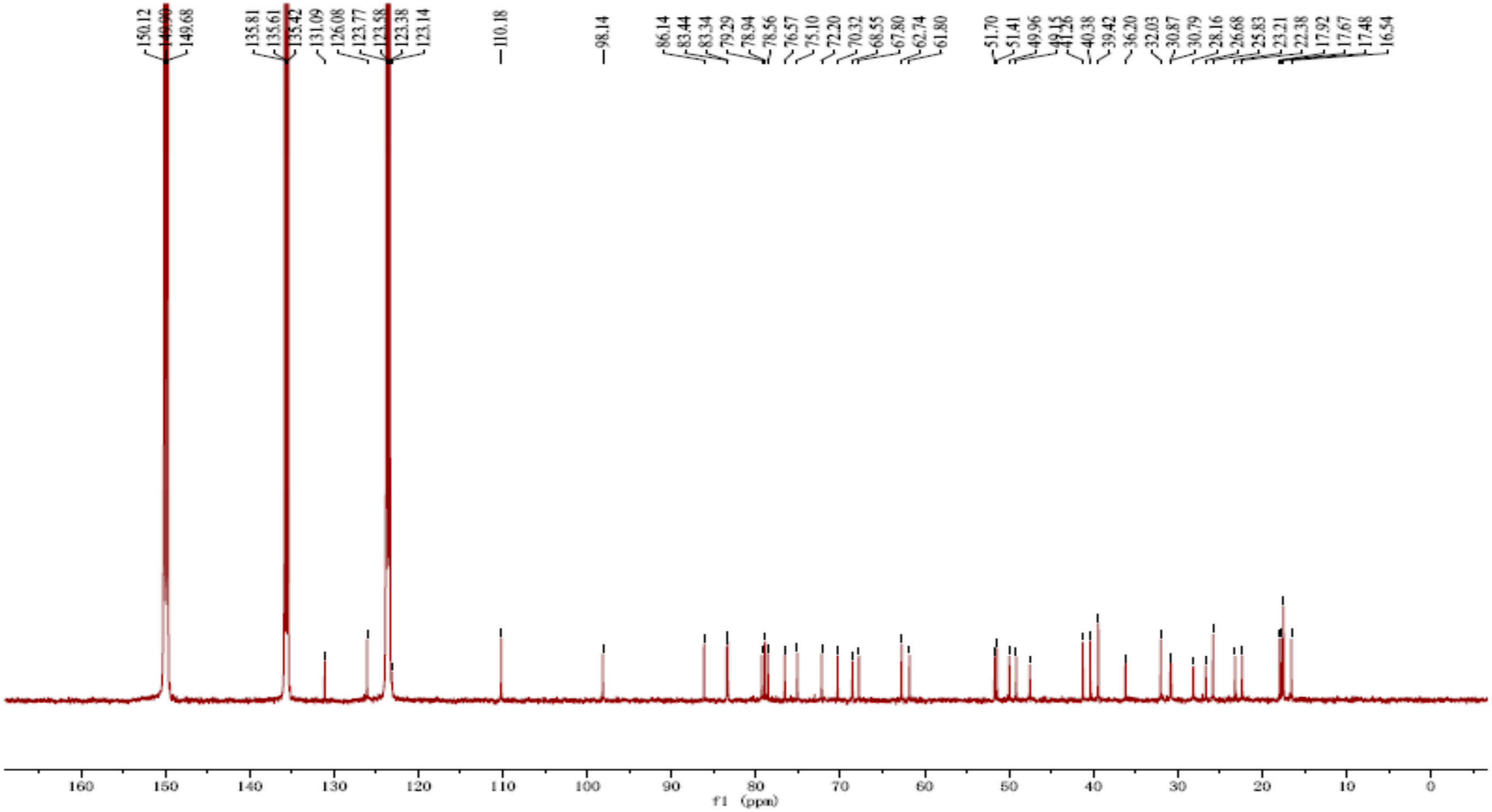


**Figure S10.** $^{13}$C-NMR spectrum of ginsenoside $F_5$ in pyridine-$d_5$.

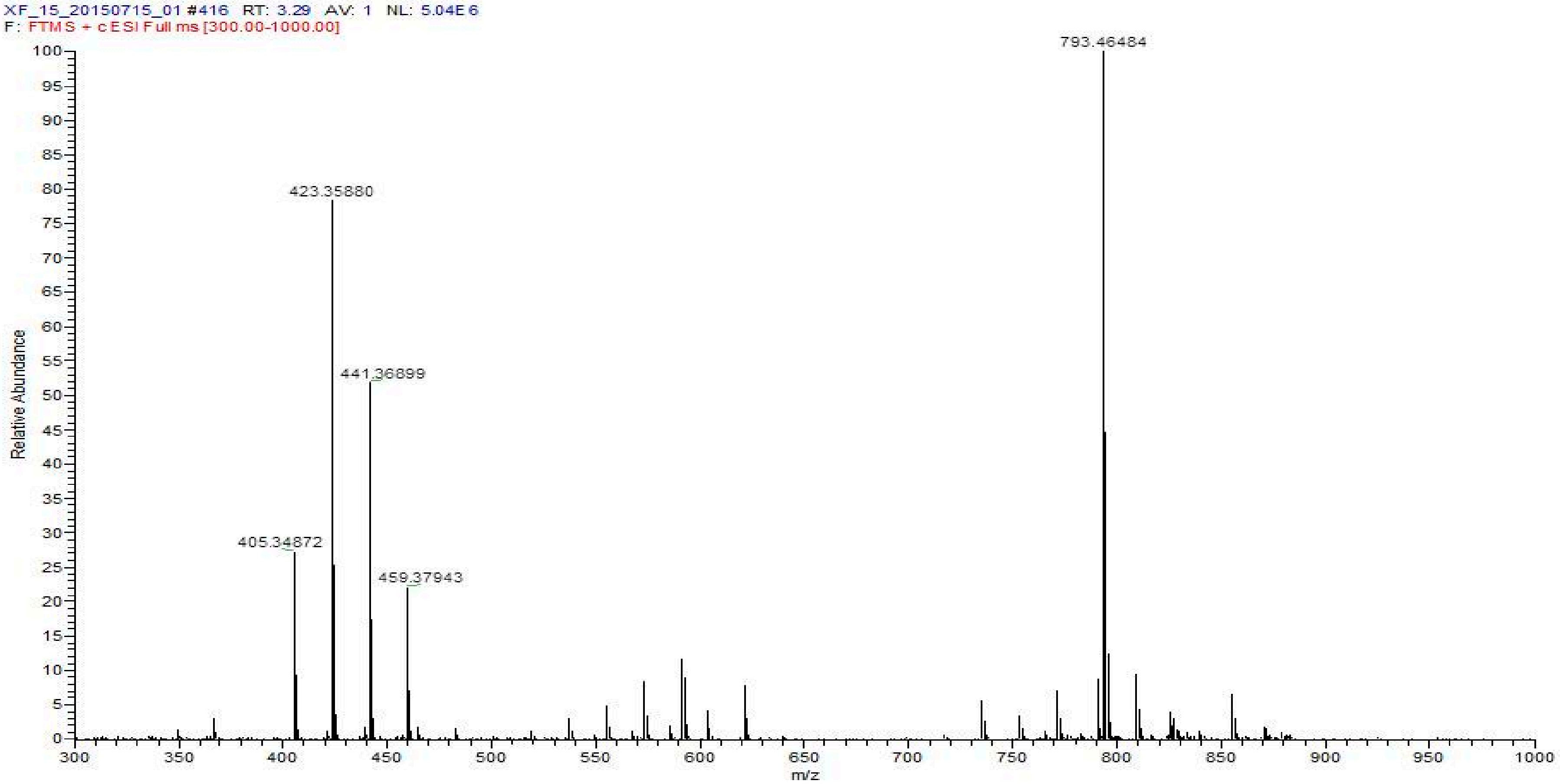


**Figure S11.** MS spectrum of ginsenoside $F_5$.

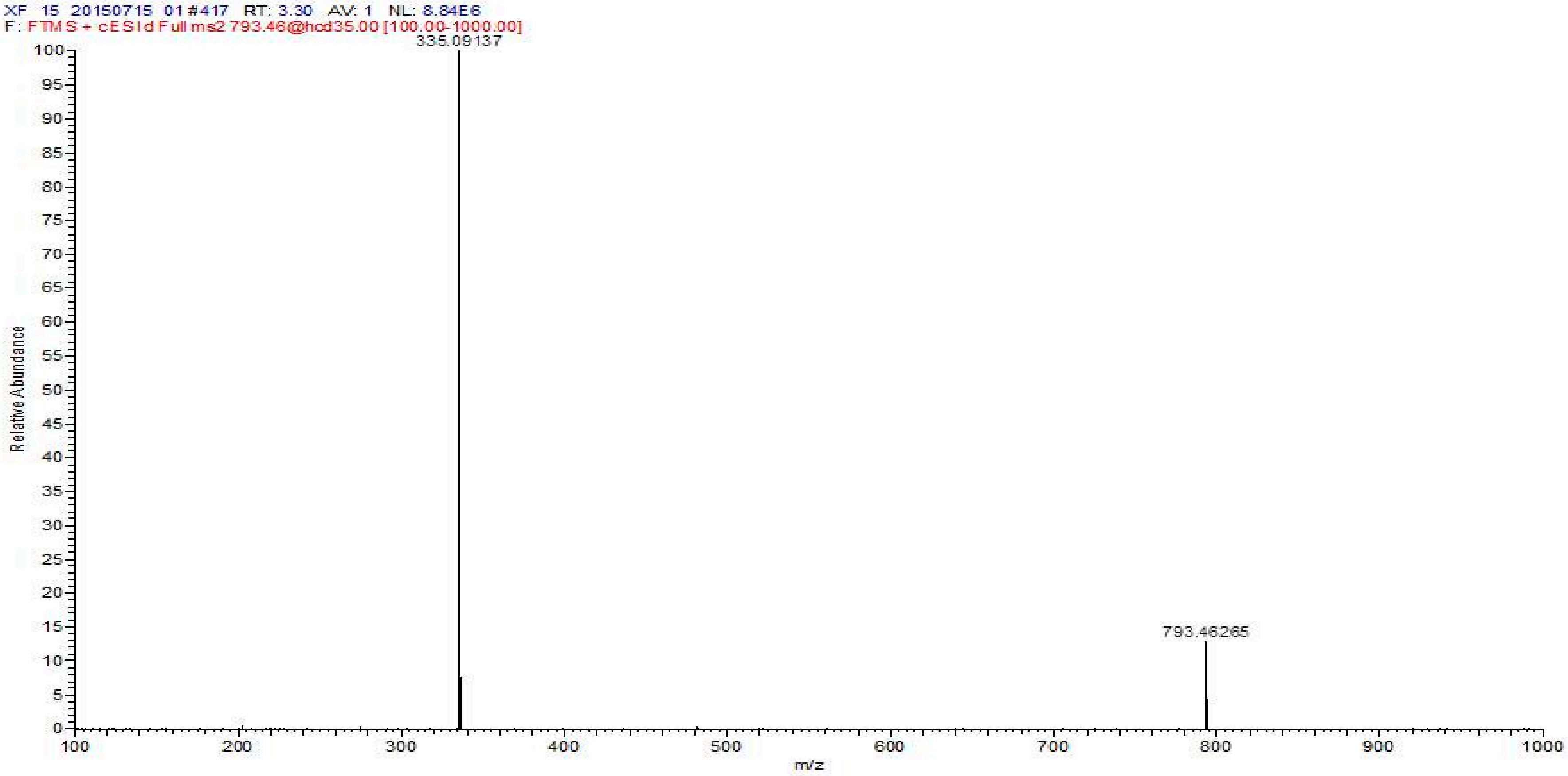


**Figure S12.** MS spectrum of ginsenoside $F_5$.

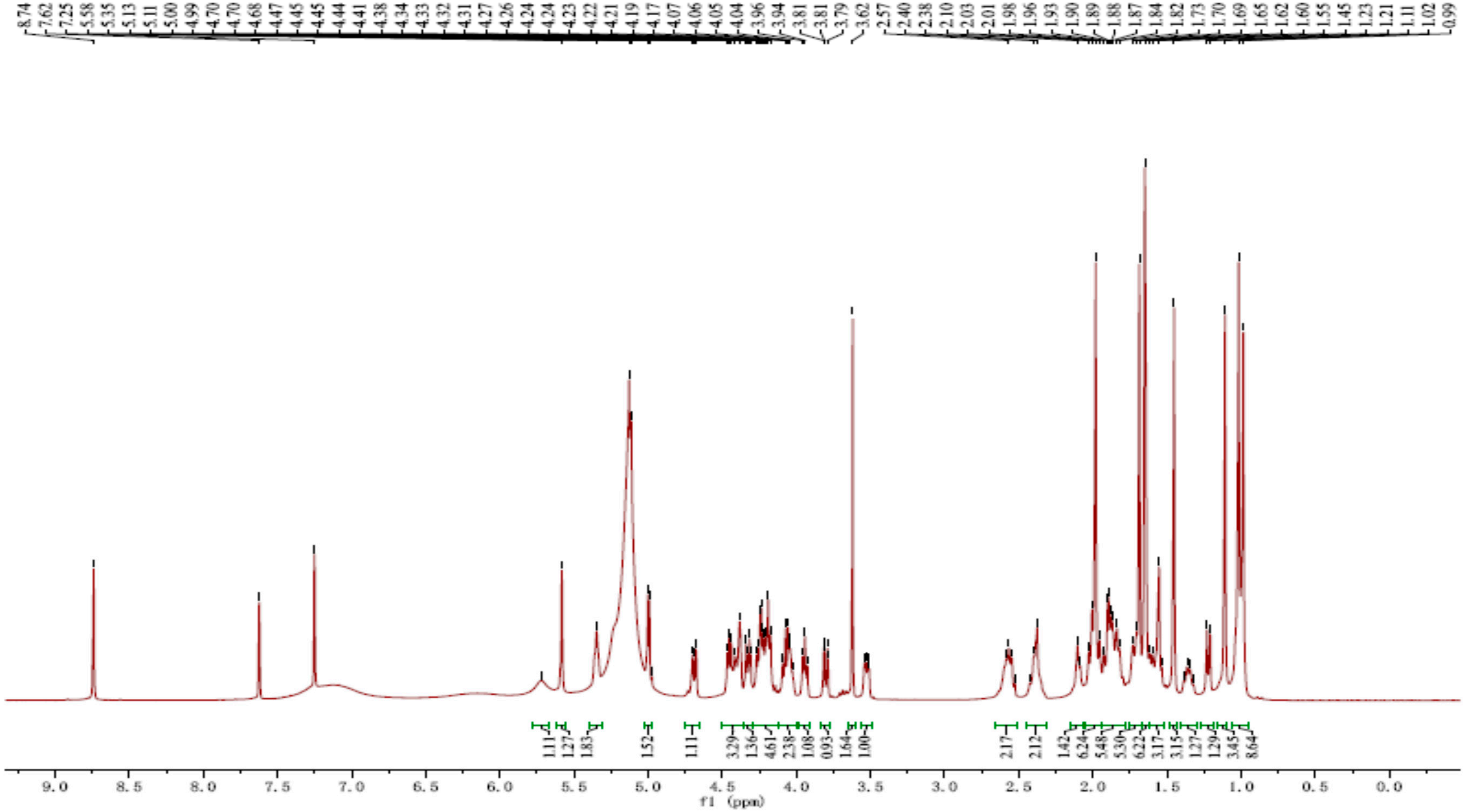


**Figure S13.** $^1$H-NMR spectrum of ginsenoside $F_3$ in pyridine-$d_5$.

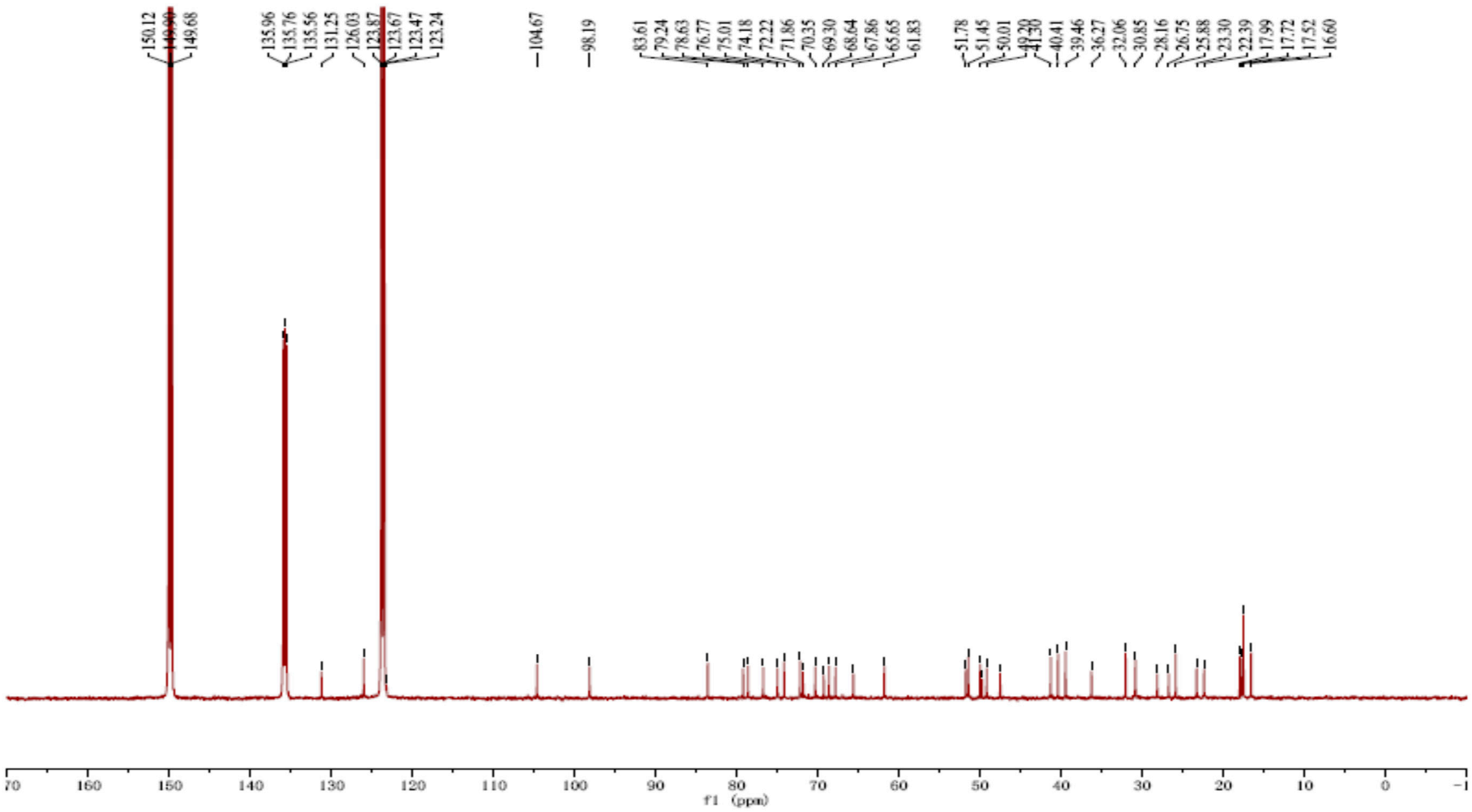


**Figure S14.** $^{13}$C-NMR spectrum of ginsenoside $F_3$ in pyridine-$d_5$.

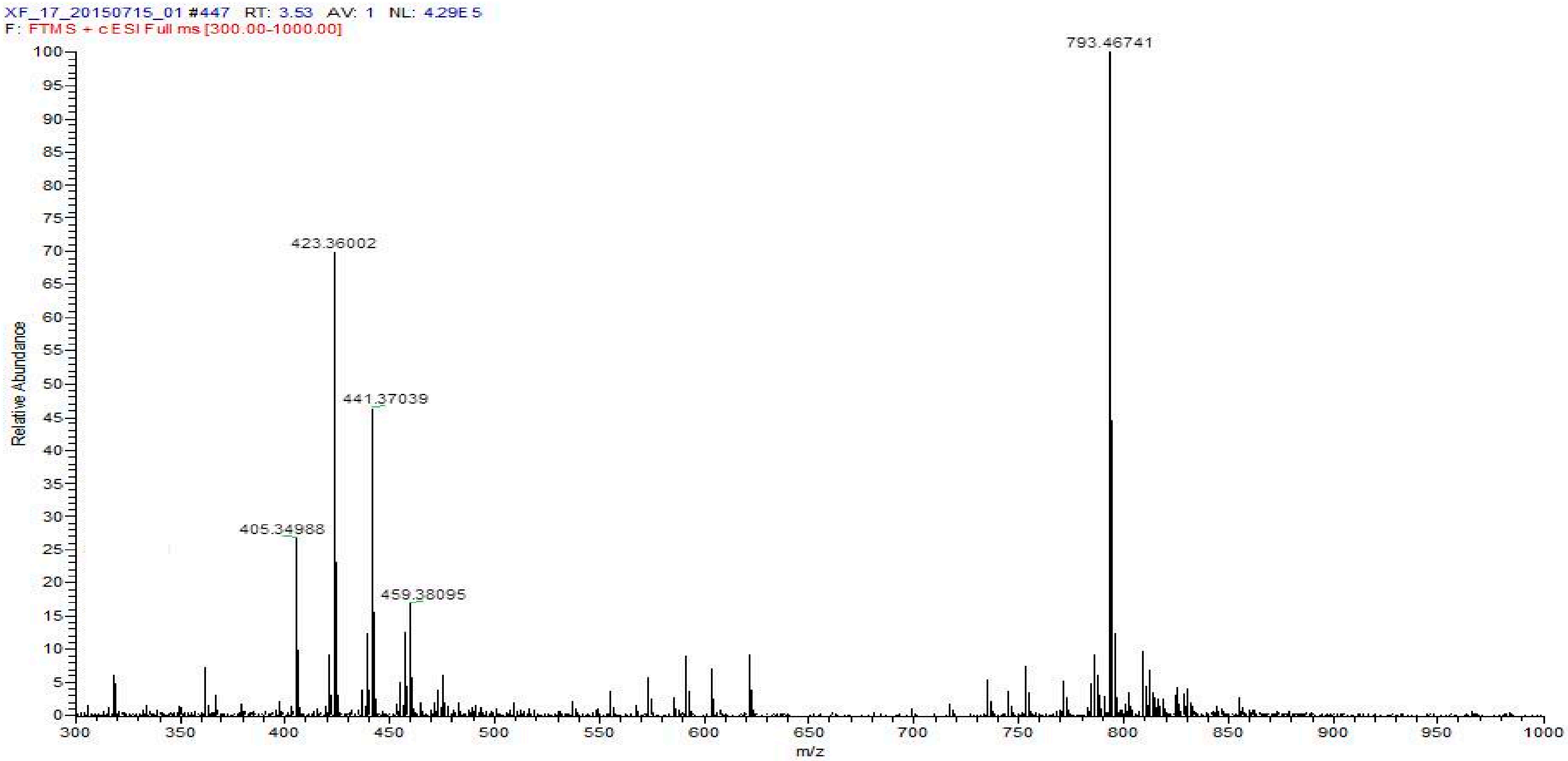


**Figure S15.** MS spectrum of ginsenoside $F_3$.

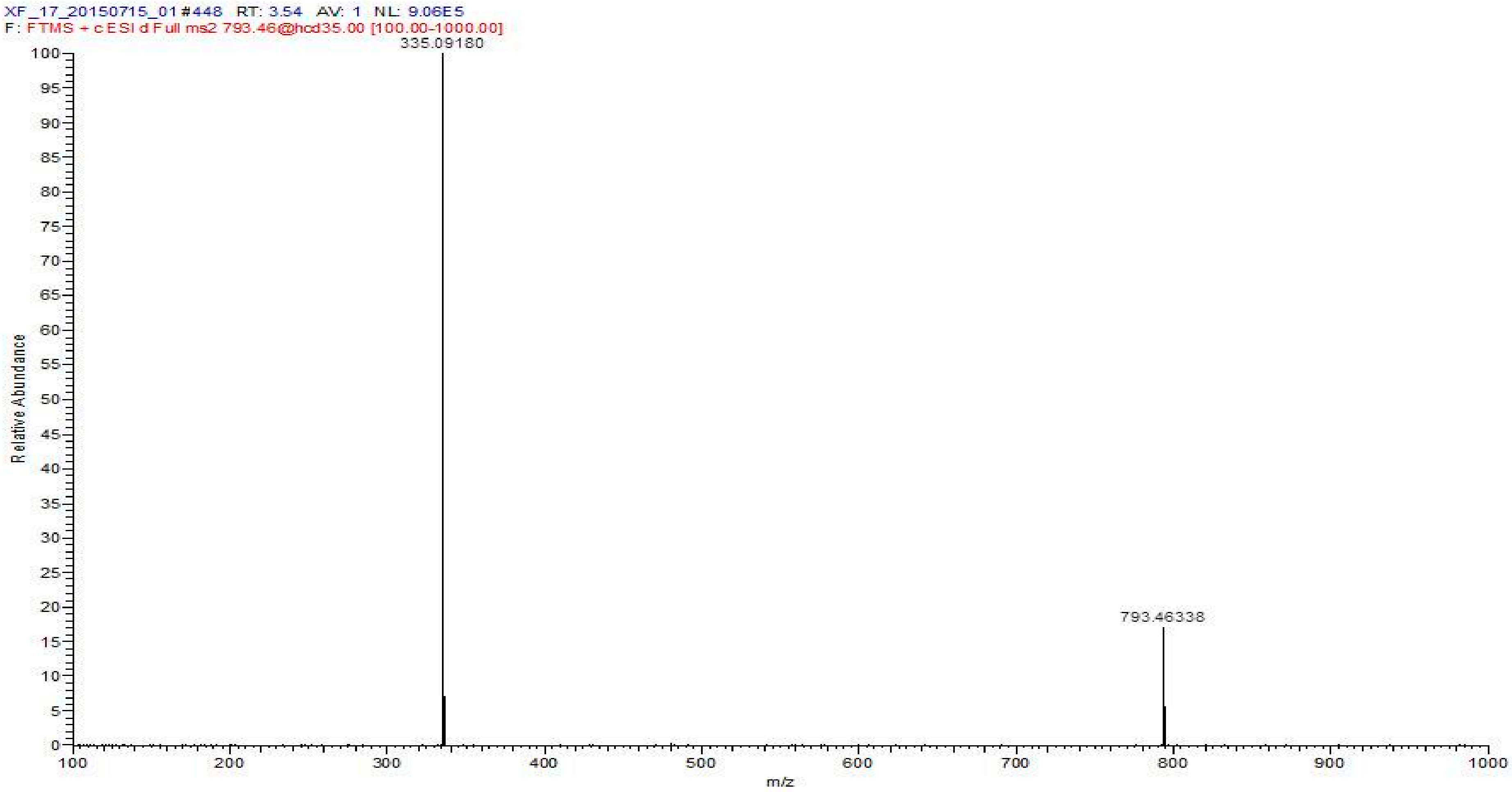


**Figure S16.** MS spectrum of ginsenoside $F_3$.
